# Notch Signaling Hydrogels Enable Rapid Vascularization and Promote Dental Pulp Tissue Regeneration

**DOI:** 10.1002/advs.202310285

**Published:** 2024-07-16

**Authors:** Siyuan Zhang, Mei Yu, Maojiao Li, Min He, Li Xie, Fangjun Huo, Weidong Tian

**Affiliations:** ^1^ State Key Laboratory of Oral Diseases National Clinical Research Center for Oral Diseases National Engineering Laboratory for Oral Regenerative Medicine Engineering Research Center of Oral Translational Medicine Ministry of Education West China Hospital of Stomatology Sichuan University Chengdu 610041 P. R. China; ^2^ State Key Laboratory of Oral Diseases National Clinical Research Center for Oral Diseases National Engineering Laboratory for Oral Regenerative Medicine Engineering Research Center of Oral Translational Medicine Ministry of Education Department of Oral and Maxillofacial Surgery West China Hospital of Stomatology Sichuan University Chengdu 610041 P. R. China

**Keywords:** angiogenesis, dental pulp regeneration, hydrogel, notch signaling, revascularization

## Abstract

Successful dental pulp regeneration is closely associated with rapid revascularization and angiogenesis, processes driven by the Jagged1(JAG1)/Notch signaling pathway. However, soluble Notch ligands have proven ineffective in activating this pathway. To overcome this limitation, a Notch signaling hydrogel is developed by indirectly immobilizing JAG1, aimed at precisely directing the regeneration of vascularized pulp tissue. This hydrogel displays favorable mechanical properties and biocompatibility. Cultivating dental pulp stem cells (DPSCs) and endothelial cells (ECs) on this hydrogel significantly upregulate Notch target genes and key proangiogenic markers expression. Three‐dimensional (3D) culture assays demonstrate Notch signaling hydrogels improve effectiveness by facilitating encapsulated cell differentiation, enhancing their paracrine functions, and promoting capillary lumen formation. Furthermore, it effectively communicates with the Wnt signaling pathway, creating an odontoinductive microenvironment for pulp‐dentin complex formation. In vivo studies show that short‐term transplantation of the Notch signaling hydrogel accelerates angiogenesis, stabilizes capillary‐like structures, and improves cell survival. Long‐term transplantation further confirms its capability to promote the formation of pulp‐like tissues rich in blood vessels and peripheral nerve‐like structures. In conclusion, this study introduces a feasible and effective hydrogel tailored to specifically regulate the JAG1/Notch signaling pathway, showing potential in advancing regenerative strategies for dental pulp tissue.

## Graphical abstract

The study introduces a Notch signaling hydrogel designed to enable rapid dental tissue pulp revascularization. This approach orchestrates a proangiogenic microenvironment by inducing specific Notch signaling, promoting angiogenesis, and interacting with Wnt signaling to facilitate odontogenesis for pulp‐dentin complex regeneration. This findings underscore the potential of the Notch signaling hydrogel in advancing regenerative strategies for dental pulp tissue.

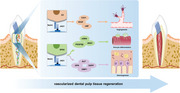

## 1 Introduction

Vascularization is essential for tissue regeneration, particularly when engineering complex tissues for which establishing sufficient microvasculature is necessary to achieve adequate oxygen and nutrient transport between cells.^[^ ^1^, ^2^ ^]^ The diffusional limit of oxygen requires cells to be within 200 µm of a capillary to meet oxygen demands.^[^ ^3^ ^]^ Therefore, significant challenges are encountered when regenerating the dental pulp, which is a highly vascularized tissue encased in within rigid mineralized surroundings with sole external access through the tooth root apex.^[^ ^4^, ^5^, ^6^ ^]^ The vascular system of the dental pulp supports the supply of nutrients and waste removal and also contributes to the inflammatory responses and the subsequent regeneration.^[^ ^7^ ^]^ Since several oral diseases lead to cause dental pulp loss, it is imperative to develop strategies for regenerating the dental pulp tissue.

Co‐engineering approaches involving cells and biomaterial substrates provides some guidance, but often fail to stimulate the desired biological responses for vascularization.^[^ ^8^, ^9^ ^]^ Modifications to synthetic biomaterials, such as adjustments in stiffness, degradability, and the presentation of matrix‐immobilized small molecules and growth factors, aim to control cell fate but lack fundamental biological insights.^[^ ^10^, ^11^ ^]^ The regeneration of vascularized tissue relies on precise coordination between different cell types, involving the regulation of specific signaling pathways to carefully control the spatial and temporal activities of the cells.^[^ ^12^, ^13^ ^]^ Therefore, enabling this intercellular coordination through the precise modulation of targeted signals is considered an optimal strategy for engineering vascularized tissues.

Notch is a conserved regulatory factor used for controlling the fate of cells, and it orchestrates the tissue morphogenic processes via the juxtracrine Notch signaling pathway.^[^ ^14^ ^]^ The significance of Notch as a key regulator of angiogenesis is the reason for it being considered a potential tool for *de novo* tissue vascularization.^[^ ^15^, ^16^ ^]^ Jagged1 (JAG1) is a ligand of Notch, which is particularly essential to angiogenesis as it interacts antagonistically with another Notch ligand named delta‐like 4 (DLL4) and thereby guides the branching and propagation of the vascular tree, often with cooperation from the vascular endothelial growth factor receptor (VEGFR) signaling.^[^ ^17^ ^]^ JAG1 drives the endothelial cells (ECs) into tip cell formation by antagonizing the DLL4/Notch1 signaling.^[^ ^18^, ^19^, ^20^ ^]^ The tip cells are located at the tips of vascular sprouts and are known to precisely regulate the process of angiogenesis.^[^ ^21^ ^]^ The process of vessel maturation is governed by JAG1‐mediated Notch activation through the promotion of the differentiation of mesenchymal stem cells (MSCs) into vascular‐supportive structures, including vascular smooth muscle cells and pericytes.^[^ ^22^, ^23^, ^24^ ^]^ In addition, JAG1 promotes dental pulp regeneration, as evidenced by its effect of inducing of odonto/osteogenic differentiation during tooth development and pulp tissue regeneration.^[^ ^25^, ^26^, ^27^ ^]^ These findings highlight that JAG1 is a suitable bioactive ligand for the regeneration of vascularized pulp tissue.

The engineering‐friendly characteristics of the Notch pathway offer valuable tools for tissue engineering,^[^ ^28^ ^]^ and the traditional approaches involve using the soluble forms of Notch ligands,^[^ ^29^, ^30^ ^]^ in vitro co‐culture of Notch receptor‐expressing cells and the cells presenting Notch ligands,^[^ ^31^ ^]^ transfection of the constitutively active forms of Notch Intracellular Domain (NICD),^[^ ^32^ ^]^ and immobilization of ligands on tissue‐culture polystyrene plates.^[^ ^26^ ^]^ Recent research has reported the use of biomaterials incorporating immobilized Notch ligands,^[^ ^33^ ^]^ considering that soluble ligands may not reliably activate Notch signaling and, therefore, do not often exhibit any activity or even antagonistic properties.^[^ ^34^ ^]^ Studies have reported the immobilization of Notch signaling ligands onto tissue engineering scaffolds, thereby directing cell‐specific responses and enhancing tissue maturation.^[^ ^22^, ^35^, ^36^ ^]^ This approach recapitulates the key developmental signaling pathways, thereby accelerating engineered tissue formation.

Notch signaling may be either amplified or attenuated to induce the differentiation of stem cells into a specific lineage for regenerative purposes. Consequently, Notch signaling scaffolds have been used in the regeneration of vairous tissues, such as vascular pericytes,^[^ ^37^ ^]^ skin,^[^ ^38^ ^]^ bone,^[^ ^35^ ^]^ liver,^[^ ^36^ ^]^ and cardiac tissues.^[^ ^39^ ^]^ Previous studies have been focused on seeding the primary cellular component of a particular tissue onto scaffolds and then culturing them to promote tissue maturation. For instance, vascular pericytes are seeded onto scaffolds and cultured in vascular tissue engineering, osteoblasts in bone tissue engineering, and cardiomyocytes in cardiac tissue engineering. Since tissues are inherently multicellular, such as vascular pericytes and ECs are present in vascular tissues, osteoblasts and osteoclasts are present in bone tissue, and cardiomyocytes and fibroblasts are present in cardiac tissues, the effect of Notch signaling scaffolds on dental pulp stem cells (DPSCs) and ECs and the roles of the scaffold in both vascularization and odontogenic differentiation processes were explored in the present study.

In this context, it was hypothesized that immobilizing JAG1 ligands on crosslinked fibrin hydrogels would promote the co‐engineering of MSCs and ECs to rapidly form capillary‐like structures. The JAG1 ligands were indirectly immobilized through protein G binding to form the JAG1‐functionalized fibrin hydrogels. The developed Notch signaling hydrogel exhibits significantly improved efficiency in facilitating engineered cell differentiation, promoting the paracrine functions of these cells, and enhancing pulp tissue revascularization and angiogenesis. Moreover, the hydrogel effectively interacted with the Wnt signaling pathway, thereby contributing to odontogenesis and creating an odonto‐inductive microenvironment for guiding the regeneration of the pulp‐dentin complex. The above findings highlighted the importance of using biofunctionalized scaffolds with signaling ligands to enhance the regeneration of vascularized tissue.

## 2 Results

### 2.1 Fabrication and Characterization of Hydrogels Functionalized with Notch Ligand JAG1

**Figure 1:**
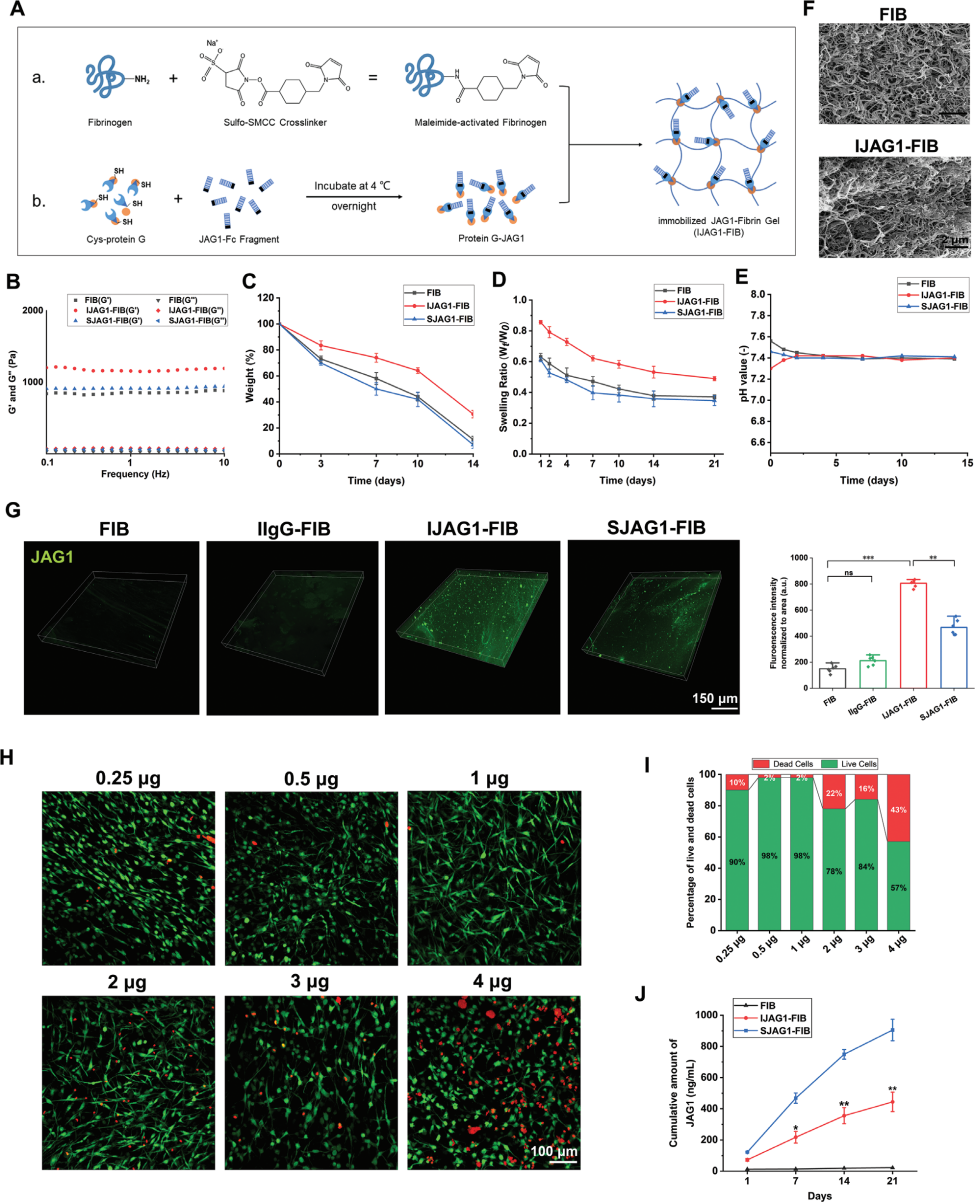
Characterization of Notch‐inducing hydrogel. A) Schematic diagram of the Notch signaling hydrogel fabrication using an indirect immobilization method. B) Rheometer measurement of storage modulus (G’) and loss modulus (G’’) of different hydrogel groups. C) Degradation, D) Swelling, and E) pH behaviors of all hydrogel groups over 14 days (n = 5). F) SEM images of fibrin gel and JAG1 immobilized fibrin gel. G) Live (green)/dead (red) staining, and I) cell viability analysis of DPSCs in hydrogels immobilized with different concentrations of JAG1 after 24‐hour culture (n = 3, by 2‐tailed Student's t‐test, **: p<0.01, ***: p<0.001, ns: non‐significant difference). J) Secretion profiles of JAG1 in hydrogels with or without JAG1 immobilization. (n = 5, by two‐tailed Student's t‐test, *: p<0.05, **: p<0.01 for IJAG1‐FIB versus SJAG1‐FIB).

An indirect mobilization method was adopted to fabricate the immobilized JAG1 fibrin gel (IJAG1‐FIB; **Figure** **1**A). In addition, the hydrogels conjugated with immobilized human IgG (IIgG‐FIB), hydrogels incorporating soluble JAG1 (SJAG1‐FIB), and the pure fibrin gel (FIB) were fabricated as control hydrogels for comparative purposes.

The stiffness of the hydrogels was varied among different groups, and the elastic modulus was measured using a rheometer. FIB exhibited an elastic shear modulus of 0.8 kPa, and upon the immobilization of JAG1 within the gel, the modulus value increased to 1.2 kPa (Figure 1B). This increase in the modulus indicated that the thiol‐maleimide Michael addition process and JAG1 conjugation enhanced the stiffness of the prepared gel. Furthermore, IJAG1‐FIB exhibited a reduced degradation rate compared to the hydrogels of the other groups, with ≈ 30% of the hydrogel remaining intact on Day 14, while the other gels had mostly degraded by this day (Figure 1C). The study of the swelling behavior of the hydrogels revealed deswelling kinetics in all groups (Figure 1D). However, IJAG1‐FIB exhibited a lower deswelling rate compared to the other gels. The pH of the surrounding solution remained stable throughout the testing period for all samples, within the physiological range of 7.3 to 7.6 (Figure 1E).

The scanning electron microscopy (SEM) analysis revealed slight crosslinking in the fibers of IJAG1‐FIB without any noticeable alterations noted in the morphology of the gels (Figure 1F). In order to quantify the immobilized JAG1, the fluorescence intensity of IJAG1‐FIB was measured and compared to that of the other gels. A significantly higher fluorescence intensity was observed for IJAG1‐FIB compared to all other hydrogels, indicating the successful immobilization of JAG1 within the fibrin gel (Figure 1G). Since the formation and maturation time of the blood vessel‐like structures and perivascular support structures is ≈ 14–21 days,^[^ ^40^ ^]^ the enzyme‐linked immunosorbent assay (ELISA) duration of 21 days was adopted. The results of ELISA indicated that the release of JAG1 was notably inhibited in IJAG1‐FIB compared to SJAG1‐FIB, which confirmed the effective immobilization of JAG1 within the fibrin gel (Figure 1J).

Further, to determine the optimal concentration of JAG1 within the fibrin gel, DPSCs were encapsulated in the hydrogels, followed by evaluation of the viability of these cells using the live and dead cells assay (Figure 1H,I). It was observed that the DPSCs remained viable in all hydrogel formulations. The gels containing 1 µg mL^−1^ JAG1 exhibited the highest percentage of viable cells, followed by the gels containing 2 µg mL^−1^ JAG1. These two samples presented a typical spindle‐like morphology, which was indicative of favorable cell adhesion and spreading. The remaining samples exhibited a relatively higher proportion of round‐shaped cells. Accordingly, it was concluded that 1 µg mL^−1^ JAG1 was the optimal concentration of JAG1 for immobilization within the hydrogel to effectively promote cell adhesion and spreading.

### 2.2 Effects of IJAG1‐FIB on 2D Cultured Cells

**Figure 2:**
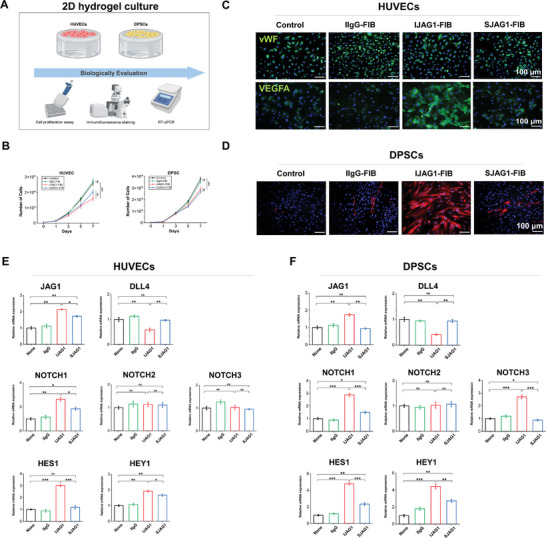
Evaluation of HUVECs and DPSCs behaviors on 2D hydrogel culture. A) Schematic diagram of biological evaluation of 2D cultured HUVECs and DPSCs on hydrogels. B) Cell proliferation analysis of HUVECs and DPSCs for 7 days, respectively. C) Immunostaining of vWF and VEGFA in HUVECs cultured on gel surface. D) Immunostaining of αSMA in DPSCs cultures on gel surface. RT‐qPCR detection of Notch signaling ligand genes (JAG1 and DLL4), receptor genes (NOTCH1, NOTCH2, NOTCH3), and target genes (HES1 and HEY1) expression in E) HUVECs and F) DPSCs, respectively. (n = 5 independent samples, by two‐tailed Student's t‐test, *: p < 0.05, **: p < 0.01, ***; p < 0.001, ns: non‐significant difference).

The effect of immobilized JAG1 on cell behavior was studied by conducting in vitro analyses to reveal any alterations in cell proliferation, cell phenotype, and Notch signaling activation in two‐dimensional (2D) cultured cells (**Figure** **2**A). The proliferative capacity of human umbilical vein endothelial cells (HUVECs) or DPSCs was assessed using the cell counting kit‐8 (CCK‐8) assay. Both cell types exhibited a significantly reduced growth rate from Day 3 when these cells were cultured on IJAG1‐FIB or SJAG1‐FIB, with no significant difference noted between the two groups (Figure 2B). In contrast, the control groups and the IIgG‐FIB groups exhibited sustained exponential growth over the 7‐day culture period. These findings indicated that JAG1 inhibited the growth of both HUVECs and DPSCs.

Next, immunostaining for the endothelial marker von Willebrand Factor (vWF) indicated no noticeable changes in EC phenotype with or without JAG1 immobilization (Figure 2C). vWF, a glycoprotein produced uniquely by ECs, is routinely used to characterize activated ECs and identify vessels in tissue sections.^[^ ^41^ ^]^ The expression of vascular endothelial growth factor A (VEGFA) was observed to have increased in HUVECs cultured on IJAG1‐FIB (Figure 2C), while no such effect was observed when the HUVECs were cultured on IIgG‐FIB or SJAG1‐FIB. Similarly, the DPSCs cultured on IJAG1‐FIB exhibited increased expression of the pericyte marker α‐smooth muscle actin (αSMA), while the lower expression of αSMA were observed in the cultured DPSCs of other groups (Figure 2D). These results suggested that immobilized JAG1 enhanced the affinity for VEGFA in HUVECs without altering their phenotype while inducing a phenotypic change in the DPSCs by upregulating the expression of αSMA.

Subsequently, cellular Notch signaling activation was assessed through reverse transcription‐quantitative polymerase chain reaction (RT‐qPCR) detection. The expression levels of the Notch signaling ligand genes, receptor genes, and target genes were determined (Figure 2E). In the HUVECs cultured on IJAG1‐FIB, JAG1 expression was significantly upregulated, while the expression of DLL4 was significantly downregulated. The Notch receptor genes, including NOTCH1, NOTCH2 and NOTCH3, and the major target genes, including HES1 and HEY1, were upregulated, indicating enhancement of Notch pathway activation. Similar results were observed for the DPSCs cultured on IJAG1‐FIB, with the Notch signaling genes significantly upregulated compared to the other groups (Figure 2F). The immunostaining results also confirmed the expression of NOTCH1 and NOTCH3 in HUVECs and DPSCs with or without JAG1 treatment (Figure S3, Supporting Information). These findings indicated that immobilized JAG1 effectively activated the Notch signaling pathway in both HUVECs and DPSCs in vitro.

### 2.3 Pro‐Angiogenic Potential of IJAG1‐FIB in Vitro

**Figure 3:**
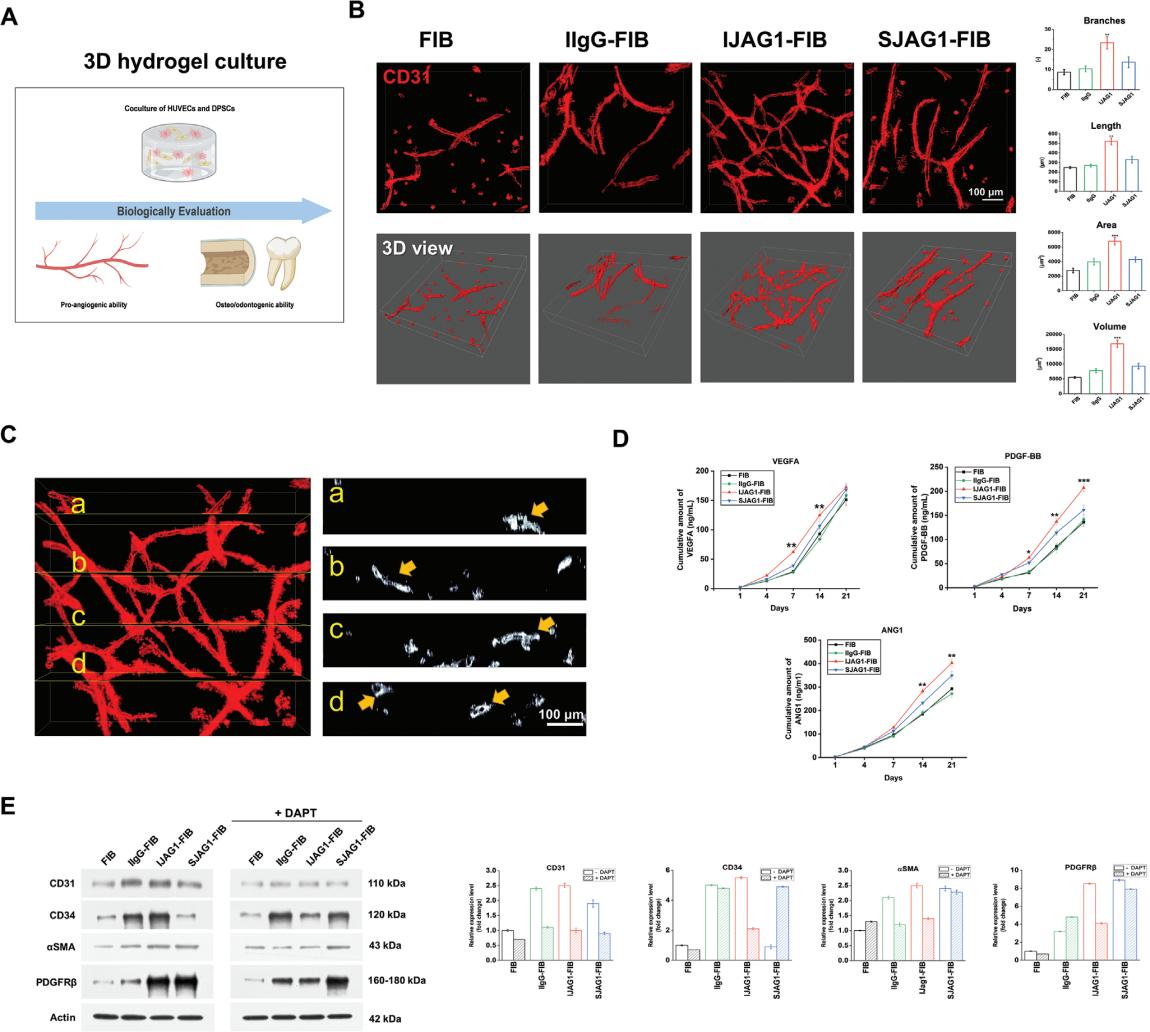
The pro‐angiogenic potential of IJAG1‐FIB in vitro. A) Schematic diagram of biologically evaluating 3D cocultured HUVECs and DPSCs in hydrogels. B) TPLSM images of CD31‐immunostained capillary‐like structures formation in different gel groups. The number of branch points, tubular length, surface area, and cell volume were measured to evaluate the quality of capillary‐like structures. C) Cross sections of the TPLSM image of IJAG1‐FIB visualize the 3D vascular networks as hollow tubular structures. (marked with yellow arrows, analyzed by Imaris software) D) ELISA analysis of the concentrations of proangiogenic factors VEGFA, PDGF‐BB, and ANG1 secreted from hydrogels. E) Western blot and quantitative analysis of protein expression for CD31, CD34, αSMA, and PDGFRβ in hydrogels with or without DAPT treatment. (n = 3 independent samples, by two‐tailed Student's t‐test, *P < 0.05, **P < 0.01, ***P < 0.001 for IJAG1‐FIB versus SJAG1‐FIB).

The effectiveness of the Notch ligand JAG1 immobilized within the fibrin gels was investigated by studying the behavior of three‐dimensional (3D) co‐cultured HUVECs and DPSCs through the evaluation of their pro‐angiogenic and osteo/odontogenic potentials within the hydrogels (**Figure** **3**A). The hydrogels loaded with DPSCs and HUVECs were subjected to immunostaining for CD31, a key endothelial marker, and the potential tubular structure formation was visualized under a two‐photon laser scanning microscope (TPLSM, Figure 3B). Capillary‐like structures were observed in all groups of hydrogels. Notably, IJAG1‐FIB presented the highest formation of tubular networks, followed by SJAG1‐FIB. In these two samples, HUVECs were aligned in a distinct direction and sprouted into 3D branched tubulars, while a few cells remained round and clustered. In contrast, the FIB and IIgG‐FIB groups had a greater number of clustered cells and fewer aligned tubular structures. Further quantification of these effects and comparison of IJAG1‐FIB with the control hydrogels were performed based on parameters such as cell volume, surface area, tubular length, and number of branch points. Significantly higher values were noted for all four parameters in the IJAG1‐FIB group, suggesting the superior potential of this group of hydrogels in promoting angiogenesis. Moreover, the cross‐sectional 3D TPLSM image stacks confirmed the maturation of capillary‐like structures, revealing the presence of lumens with diameters ranging from 5 to 20 µm (Figure 3C).

The release characteristics of pro‐angiogenic factors were evaluated using ELISA (Figure 3D). The IJAG1‐FIB groups exhibited a substantial increase in the release of VEGFA in the early stage and a significant increase in the release of platelet‐derived growth factor BB(PDGF‐BB) and angiopoietin 1 (ANG1) in the late stage compared to the other groups. VEGFA secreted from fibroblasts is the most important pro‐angiogenic factor in the early stage of angiogenesis. PDGF‐BB and ANG1 are critical mediators involved in the chemotaxis of pericytes and the growth and stabilization of vascular structures. Therefore, the above results further substantiated that IJAG1‐FIB enabled the rapid formation of mature vascular structures.

Consistent with the above results, the hydrogels functionalized with the Notch ligand JAG1 exhibited a higher expression of CD31, as evidenced by the results of western blotting (Figure 3E). This upregulation of CD31 expression was observed in response to JAG1 immobilization. After treatment with N‐[N‐(3,5‐difluorophenacetyl)‐L‐alanyl]‐S‐phenylglycinet‐butyl ester (DAPT), which is a Notch signaling inhibitor, effective abrogation of the JAG1‐induced upregulation of CD31 expression was noted. IJAG1‐FIB also exhibited increased expression of the pericyte marker αSMA, and this effect was attenuated by the DAPT treatment. These results indicated that JAG1 immobilization within the hydrogels enhanced the expression of CD31 in HUVECs and αSMA in pericytes, indicating the promotion of angiogenesis and vascular stabilization. These effects were mediated by Notch signaling activation, as evidenced by the inhibitory effect of DAPT.

### 2.4 Osteogenic and Odontogenic Differentiation Capability of IJAG1‐FIB in Vitro

**Figure 4:**
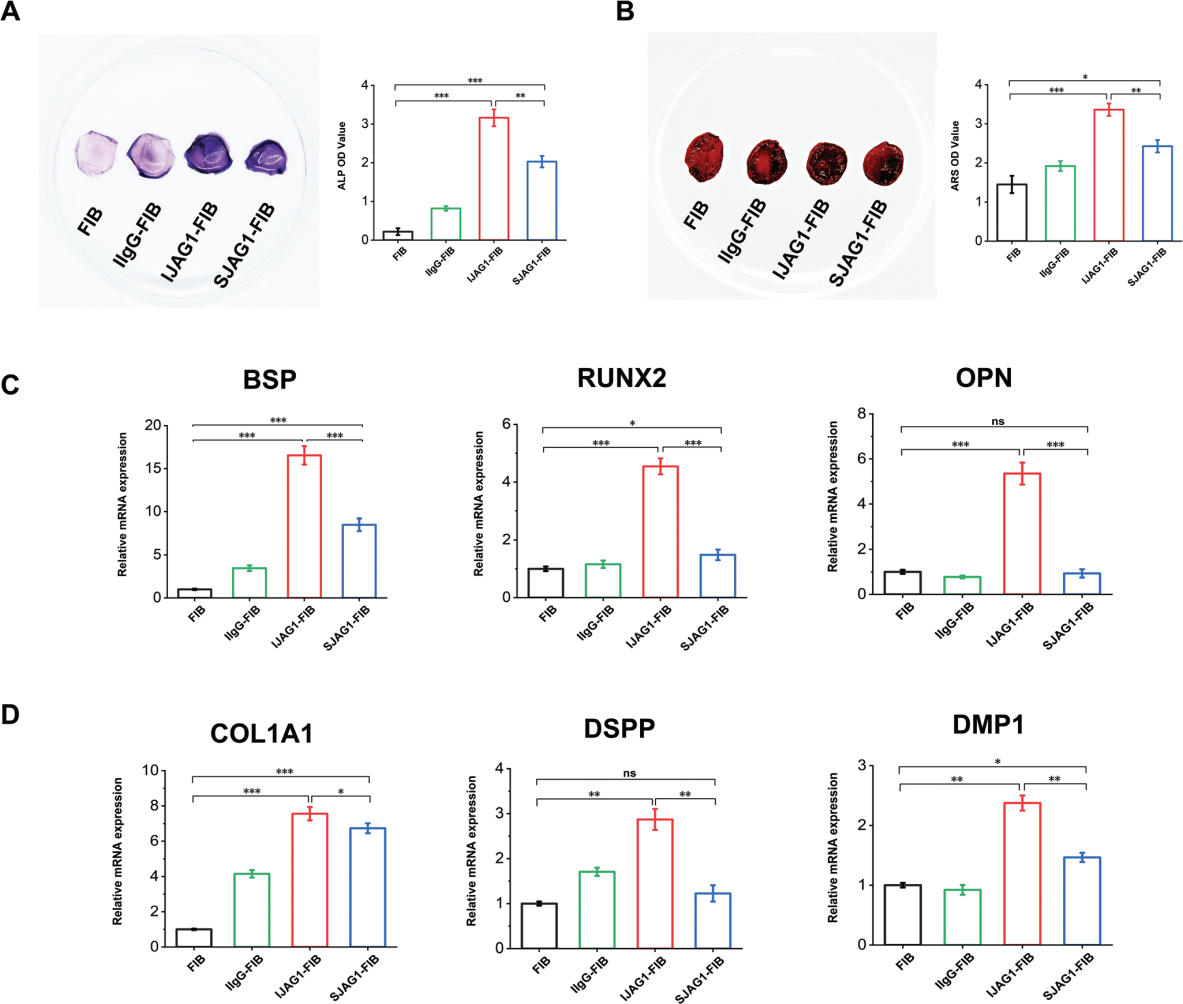
Osteogenic and odontogenic differentiation capability of IJAG1‐FIB in vitro. A) ALP staining and quantification of hydrogels after 7‐day culture in osteogenic medium. B) ARS staining and quantification of the mineralized matrix in hydrogels after osteoinduction for 14 days. C) RT‐qPCR detection of osteogenic marker genes BSP, RUNX2, and OPN expression. D) RT‐qPCR detection of odontogenic marker genes COL1A1, DSPP, and DMP1 expression. (n = 5 independent samples, by two‐tailed Student's t‐test, *: P < 0.05, **: P < 0.01, ***: P < 0.001, ns: non‐significant difference).

The osteogenic/odontogenic differentiation capacity of IJAG1‐FIB was evaluated by culturing cell‐laden hydrogels in an osteo‐induction medium for 7 days and 14 days, followed by alkaline phosphatase (ALP) and Alizarin Red S (ARS) staining. In the results of both staining analyses, the IJAG1‐FIB groups exhibited higher ALP activity (**Figure** **4**A,B) and retained enhanced mineralization capability compared to the other groups. These results were confirmed in the quantitative analysis as well. The RT‐qPCR analysis of the osteogenic marker genes bone sialoprotein (BSP), runt‐related transcription factor 2 (RUNX2), and osteopontin (OPN) (Figure 4C) and that of the odontogenic marker genes collagen type I alpha‐1 chain (COL1A1), dentin sialophosphoprotein (DSPP) and dentin matrix protein 1 (DMP1) (Figure 4D) further confirmed that IJAG1‐FIB promoted the formation of mineralized tissues and odontoblast‐like lineage differentiation. Collectively, these data suggested that IJAG1‐FIB retained robust osteogenic and odontogenic capabilities in vitro.

### 2.5 Ectopic Rapid Vascularization Capacity of IJAG1‐FIB in Vivo

**Figure 5:**
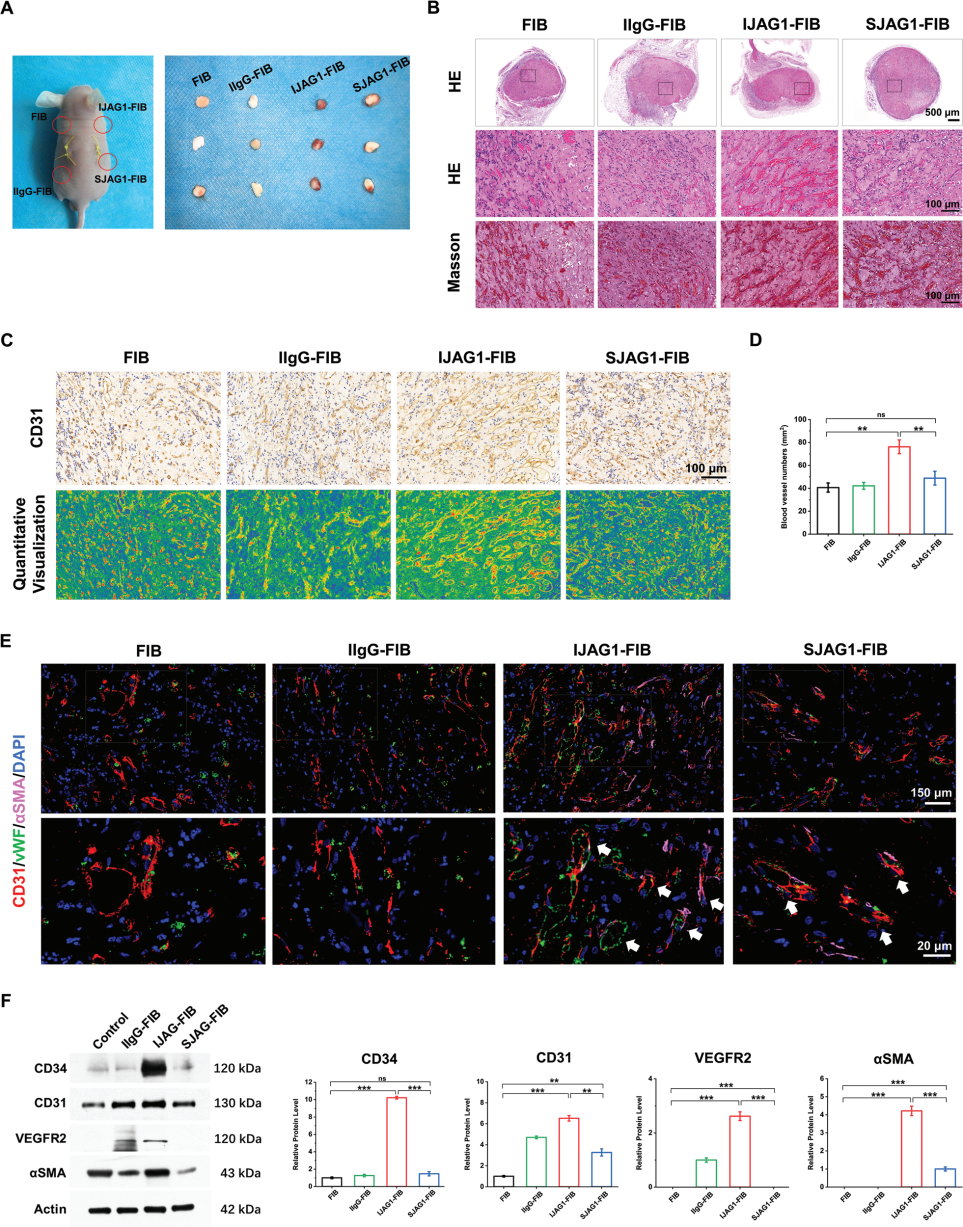
Ectopic rapid vascularization capacity of IJAG1‐FIB in vivo. A) Subcutaneous implantation sites of gel groups under the dorsal skin of nude mice (left), followed by harvest of gel groups after 5 days (right). B) H&E and Masson staining of the harvested samples. C) Immunohistochemical staining for CD31 and the CD31‐positive area was quantitatively visualized with signal intensity represented as low (green), medium (yellow), and high (red). D) Quantitative analysis of CD31‐positive blood vessels and the vessel numbers were normalized to the tissue area (mm^2^). E) Immunofluorescence triple staining to visualize the blood vessel formation (CD31, vWF, and αSMA). F) Western blot and quantitative analysis of protein expression for endothelial markers CD31, CD34, VEGFR2, and pericyte marker αSMA. (n = 5 biologically independent samples, by two‐tailed Student's t‐test, *: P < 0.05, **: P < 0.01, ***: P < 0.001, ns: non‐significant difference).

In order to better understand the effects on angiogenesis, IJAG1‐FIB was transplanted into the dorsum of nude mice, and its effects in vivo were compared to those of the other hydrogels. On Day 5 after the implantation, IJAG1‐FIB was visualized to have effectively induced neo‐vessel formation (**Figure** **5**A). Numerous new blood vessels with red blood cells, were observed after hematoxylin and eosin (H&E) staining (Figure 5B). The CD31 staining analysis confirmed the presence of ECs and quantified the neo‐vessel formation (Figure 5C). The quantitative image analysis (Figure 5D) revealed a significant increase in the number of blood vessels in the IJAG1‐FIB groups in compared to the control groups.

The degree of maturity of forming the vessel network was further assessed by triple‐stainning the hydrogels for CD31, vWF, and αSMA (Figure 5E). The IJAG1‐FIB group exhibited a greater abundance of both CD31 and vWF vessels compared to the control groups. The pericyte marker αSMA was lined surrounding the CD31 and vWF‐positive vessel walls, indicating that the peri‐capillary pericytes resided in the regenerated tissues. The results of western blotting further demonstrated that IJAG‐FIB was enriched in the endothelial markers CD31, CD34, and VEGFR2 and the pericyte marker αSMA (Figure 5F). In summary, IJAG1‐FIB was demonstrated to successfully induce rapid vascularization and promote the maturation of caplillary‐like structures.

### 2.6 IJAG1‐FIB Enabled Regeneration of Pulpo‐Dentinal Complex‐Like Tissues in Vivo

**Figure 6:**
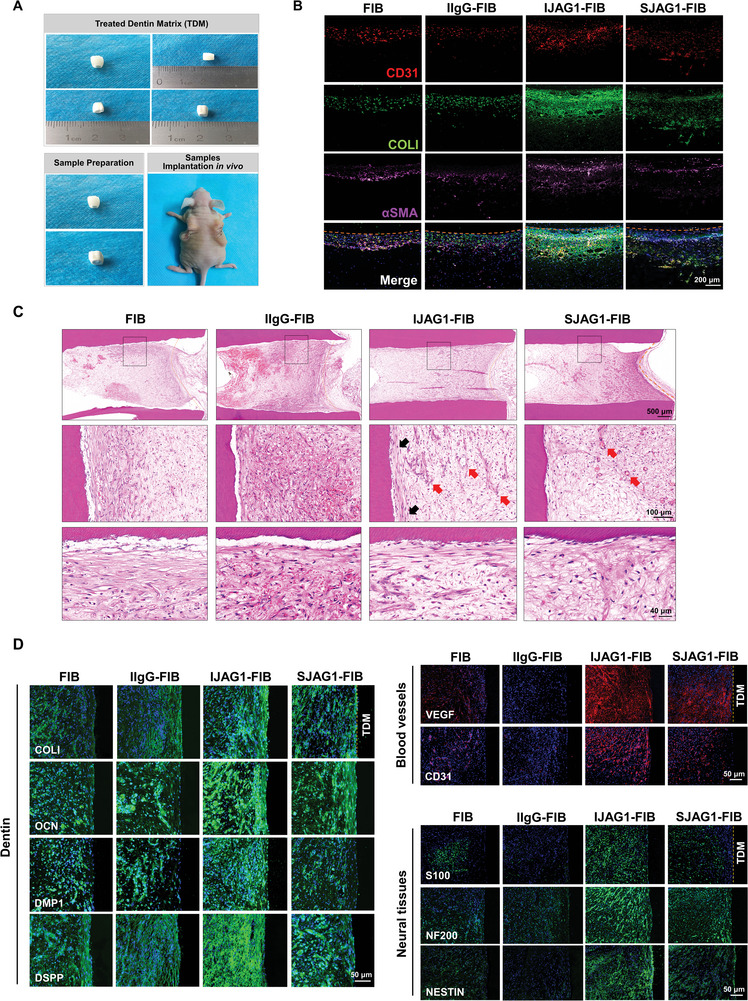
IJAG1‐FIB enabled the regeneration of pulpo‐dentinal complex‐like tissues in vivo. A) TDM preparation and TDM fill with gel samples subcutaneous implantation. B) Immunofluorescence triple staining to visualize the blood vessel formation (CD31, COL1, and αSMA) at 2 weeks of subcutaneous implantation. C) H&E staining of hydrogels harvested at 6 weeks of subcutaneous implantation. A pulpo‐dentinal complex‐like structure was formed only in the IJAG1‐FIB. Blood vessels are indicated using red arrows. Odontoblast‐like cells are indicated using black arrows. D) Immunofluorescence staining for osteo/odontogenic markers (COL1, OCN, DMPA, and DSPP), blood vessel‐specific markers (VEGF and CD31), and neural tissue‐specific markers (S100, NF200, and NESTIN).

Whether IJAG1‐FIB would facilitate the regeneration of further complex tissues, such as the pulpo‐dentinal complex, was investigated next. Cell‐laden IJAG1‐FIB, SIJAG1‐FIB, IIgG‐FIB, and FIB were inserted into treated dentin matrix (TDM) and then subcutaneously transplanted into immunocompromised mice, which were then monitored for 2 to 6 weeks (**Figure** **6**A).

After 2 weeks of implantation, the IJAG1‐FIB group exhibited more distinct vessel structures and increased the CD31‐positive vessel density in compared to the other groups (Figure 6B). The extracellular matrix with highly expressed Collagen I was also noted in the IJAG1‐FIB group. These results suggested that IJAG1‐FIB facilitated early‐stage neovascularization in the regenerated pulp tissues.

After 6 weeks of implantation, regenerated pulpo‐dentinal complex structures were observed in all four groups of mice. However, IJAG1‐FIB exhibited a greater density of vessels in the middle part of the regenerated tissue (Figure 6C, indicated using red arrows). The regenerated tissue in IJAG1‐FIB was homogeneous and had pulp‐like connective tissue structures with several layers of odontoblast‐like cells located on the outermost shell of the pulp‐like tissues (Figure 6C, indicated using black arrows). The biological safety of all groups of hydrogels was investigated (Figure S4, Supporting information). The major organs in nude mice were retrieved and dissected, and no noticeable changes in the organs were noted among the different groups.

The newly formed tissue by IJAG1‐FIB was further characterized through the immunostaining of dentin‐related markers DMP1 and DSPP and the vascularization‐related markers VEGF and CD31 (Figure 6D). In all cases, robust immunofluorescence signals were detected in the IJAG1‐FIB groups. Interestingly, IJAG1‐FIB increased innervation with enhanced expressions of neural markers S100, NF200, and NESTIN. The other gel groups presented no such effects. These findings suggested that the JAG1 immobilized within the hydrogel exerted synergistic effects. Nestin staining further confirmed the presence of odontoblast‐like cells within IJAG1‐FIB and also distinguished these cells from the osteoblast‐like cells that also express osteocalcin (OCN).^[^ ^42^ ^]^ Collectively, these results suggested that IJAG1‐FIB facilitated the efficient regeneration of the pulpo‐dentinal complex‐like tissue in vivo, while FIB, IIgG‐FIB, and SJAG1‐FIB could not.

### 2.7 Transcriptome Analysis Identified the Signature Gene Network in IJAG1‐FIB

**Figure 7:**
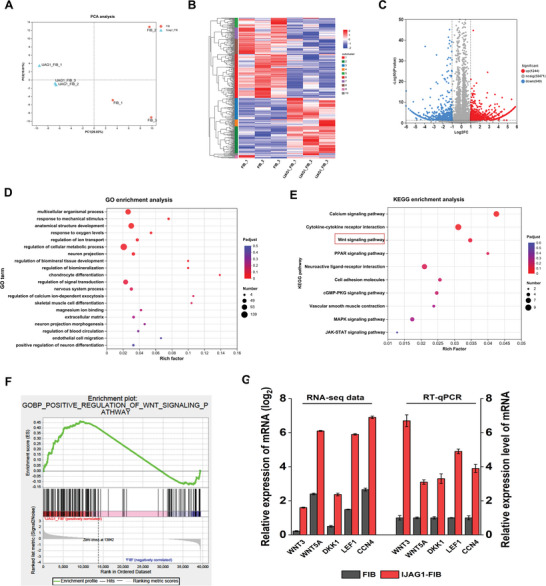
Transcriptome analysis identified the signature gene network in IJAG1‐FIB. A) Principal components analysis of IJAG1‐FIB and FIB samples. B) Heat map of the differentially expressed genes in IJAG1‐FIB compared to FIB. C) The volcano plots of differentially expressed genes. D) The top 20 significantly enriched GO biological processes in IJAG1‐FIB were shown. E) Top 10 significantly enriched signaling pathway were shown. F) GSEA results revealed that IJAG1‐FIB up‐regulates the Wnt signaling pathway. G) Confirmation of Wnt signaling pathway genes by RT‐qPCR. RT‐qPCR analysis was performed for selected candidate genes enriched in IJAG1‐FIB, including WNT3, WNT5A, DKK1, LEF1, and CCN4. (n = 3 independent samples).

The potential molecular mechanism that led to the observed functional differences between IJAG1‐FIB and FIB was explored through a transcriptome analysis of these two groups of cells. The principal components analysis indicated that the IJAG1‐FIB groups were significantly different from the FIB group, and this finding was consistent with the observed phenotype in vitro and in vivo (**Figure** **7**A). Significantly, the differentially expressed genes (DEGs) in these cells were also identified, and these genes were clustered into ten groups. Five of these ten groups, namely group III, group IV, group II, group IX, and group X represented the genes highly enriched in IJAG1‐FIB. These five groups were the most interesting groups. The remaining five groups represented the genes highly expressed in FIB (Figure 7B). The volcano plot of all the detected genes revealed that the expression levels of 2193 genes were significantly altered (> 2‐fold, p < 0.05) between the IJAG1‐FIB and the control group, with 1244 genes upregulated and 949 genes down regulated (Figure 7C). The gene ontology (GO) analysis revealed a significant enrichment of these genes in tissue development, and the top 20 significantly enriched GO biological processes revealed for IJAG1‐FIB indicated that the upregulated genes were associated with biomineralization, angiogenesis, and neuron differentiation (Figure 7D). In order to reveal the genes with insignificant differential expression and significant biological significance, Gene Set Enrichment Analysis (GSEA) was performed by co‐analyzing the IJAG1‐FIB and FIB DEG sets and 1330 standardized GSEA molecular feature database gene sets. As depicted in Figure S5 (Supporting information), the three gene sets POSITIVE_REGULATION_OF_SPROUTING_ANGIOGENESIS, ODONTOGENESIS_OF_DENTIN_CONTAINING_TOOTH, and POSITIVE_REGULATION_OF_NEUROGENESIS were enriched, which is consistent with the observed phenotypes in vivo.

Further, the signaling pathway analysis indicated that the genes involved in the Wnt, peroxisome proliferator‐activated receptor (PPAR), and mitogen‐activated protein kinase (MAPK) signaling pathways were also enriched in IJAG1‐FIB (Figure 7E). Since the Wnt signaling pathway plays an important role in odontogenesis, it was particularly focused on in the subsequent analysis. The GSEA results confirmed that compared to FIB, IJAG1‐FIB exhibited the enrichment of a greater number of gene sets that positively regulated Wnt signaling (Figure 7F). The top 5 enriched genes in Wnt signaling were listed. The expression levels of these enriched genes were evaluated using RT‐qPCR analysis, and the results were consistent with the sequencing data, suggesting that the dataset obtained from transcriptome analysis accurately reflected the gene expression differences between FIB and IJAG1‐FIB (Figure 7G). In summary, the transcriptome analysis identified the genes involved in Wnt signaling that were specifically expressed in IJAG1‐FIB.

## 3 Discussion

Successful pulp regeneration relies on the establishment of an angiogenesis‐friendly environment for rapid revascularization. Therefore, the present study targeted enhanced dental pulp regeneration by incorporating a controlled signal ligand into a scaffold to create a conducive microenvironment for targeted cell differentiation. This was achieved by fabricating a Notch signaling hydrogel with immobilized JAG1 using the indirect immobilization approach that enhanced the activation of the Notch signaling pathway, promoting sustained cell viability and angiogenesis. In addition, Wnt signaling was revealed to play a synergistic role during this process and further aided dental pulp tissue regeneration and mineralization. The beneficial effects of Notch on MSC behavior have already been studied previously in the context of tissue regeneration, although in a different way, while the Notch signaling gel prepared in the present study served as the first platform to elucidate the angiogenic mechanisms, thereby having immense value in the field of biomaterials and regenerative medicine.

The hydrogel system prepared in the present study was optimized for stiffness, with an elastic modulus of 1.2 kPa, coupled with JAG1 stimulation, which facilitated the efficient angiogenesis of cells in a 3D environment. The stiffness optimization results were consistent with the literature, which also reports that 1–2 kPa is the optimal matrix stiffness for blood vessels formation.^[^ ^43^ ^]^ The present investigation focused on tracing the progressive changes in MSC and EC behaviors, transitioning from the 2D culture surface to the 3D inner matrix. Consistent with previous studies, JAG1 was demonstrated to inhibit MSC and EC proliferation while enhancing the differentiation potential of both cell types.^[^ ^44^, ^45^ ^]^ In monoculture, both cell types were observed to exhibit JAG1‐mediated angiogenic activities in vitro. DPSCs demonstrated pericyte differentiation, while HUVECs expressed proangiogenic molecules. These findings suggested that the Notch signaling hydrogel promoted the dominance of pericyte‐like properties in DPSCs and mature EC‐like properties in HUVECs upon immobilized JAG1 treatment. These characteristics became further pronounced during co‐culture in the 3D Notch‐inducing environment, which was attributed to the following reasons: (i) the formation of capillary‐like structures was superior compared to the gels without Notch signaling (ii) vessel stabilization due to the presence of pericytes was more significant; (iii) the increased production of pro‐angiogenic molecules was noted in the early and late stages in the Notch signaling gels, as evidenced by the results of ELISA, RNA‐sequencing, and RT‐qPCR. The pericyte‐like DPSC and mature EC‐like HUVEC activities were further validated through an in vivo assay involving ectopic short‐term subcutaneous transplantation and the gel plug assays. Accordingly, this advanced functional material platform is expected to address the concerns related to rapid revascularization and angiogenesis, ensuring tissue regeneration.

Further, the fate of MSCs and ECs in the defined pro‐angiogenic niche with the engineered micro‐capillary networks induced by the synthetic Notch signaling hydrogels was studied. The MSCs demonstrated the ability to support the EC functions during micro‐capillary formation within the natural ECM hydrogel materials in addition to modulating their own microenvironment and providing the ECM.^[^ ^46^, ^47^ ^]^ These findings were consistent with previous studies and indicated that capillary formation is favored at low hydrogel stiffness and material density. Moreover, the levels of the well‐known pro‐migratory and pro‐angiogenic factors VEGF‐A, PDGF‐BB, and ANG1 increased significantly during the micro‐capillary network formation, and this increase was dependent on JAG1 immobilization. This finding suggested that the micro‐capillary network assembly was the most efficient when the culture conditions best supported cell migration, cell communication, and matrix remodeling‐related essential functions during the native vascular processes. Interestingly, DAPT treatment significantly disrupted ithe JAG1‐induced angiogenesis in IJAG1‐FIB, while little influence was noted in the case of the other gels. This observation demonstrated the specific JAG1‐induced angiogenesis in Notch‐signaling hydrogels. Activation of the Notch pathway in the perivascular cells occurs through the interaction of the transmembrane protein Notch with the EC surface‐presented JAG1.^[^ ^18^ ^]^ Notably, JAG1, as a transmembrane protein, has previously been demonstrated to contribute to cell fate decisions through cell–cell communication, acting either as an agonist or antagonist for Notch signaling when presented in immobilized or soluble forms, respectively.^[^ ^48^ ^]^ According to the above findings coupled with the observations from previous studies, it was inferred that ECs stimulate the Notch target genes in perivascular MSCs in a JAG1‐dependent manner, thereby contributing to the perivascular commitment of these cells. Indeed, the present study pioneered to demonstrate that Notch‐inducing biomaterials trigger rapid angiogenesis during tissue regeneration.

In addition, subsequent activation of the Wnt pathway was noted in the Notch signaling gels, which highlighted the cooperative relationship of Notch signaling with Wnt/β‐Catenin signaling, which is known to promote osteo/odontogenesis. The present study demonstrated that the JAG1 peptide‐induced Notch signaling enhances Wnt/β‐catenin signaling, thereby exerting a synergistic effect on the osteogenesis and odontogenesis of MSCs both in vitro and in vivo. In order to further investigate this synergistic effect, the downstream markers of the Wnt/β‐catenin pathway, including WNT3 and WNT5A, were analyzed. The RT‐qPCR results revealed a significant upregulation of both WNT3 and WNT5A expressions in the JAG1 immobilization group compared to the control groups, providing robust evidence in support of the above‐stated hypothesis regarding the synergistic effect. The Wnt/β‐catenin signaling pathway reportedly plays a multifaceted role in tooth morphogenesis.^[^ ^49^ ^]^ Previous studies have also indicated that the Wnt/β‐catenin signaling plays an important role in angiogenesis,^[^ ^50^ ^]^ odontoblastic differentiation,^[^ ^51^ ^]^ and neural differentiation^[^ ^52^ ^]^ in dental mesenchymal stem cells, while serving as an important signal for enhancing the regeneration of the pulp‐dentin complex. Therefore, in the context of signaling pathways, the present study demonstrated that the Notch signaling hydrogel induces differentiation, angiogenesis and odontogenesis synergistically, potentially through the activation of pivotal signaling pathways, such as Wnt/β‐catenin, thereby providing the possibility for the restoration of the functional pulp‐dentin complex.

The trophic effect is recognized to be essential for effective coordination among different cell types within tissues. Traditionally, this effect is mediated through signaling by secreted soluble factors, thereby facilitating communication between neighboring cells in a paracrine manner. In the present study, the trophic effect observed in the JAG1‐immobilized hydrogel supported the regeneration of the pulp‐dentin complex by promoting rapid angiogenesis and the subsequent activation of Wnt signaling, thereby enhancing the activity of the engineered cells. Notch is a conserved signaling pathway factor involved in the regulation of cell proliferation, cell death, and cell fate differentiation in a broad range of developmental processes. Therefore, the Notch signaling hydrogels serve as a platform to further explore the role of perivascular niches in tissue regeneration. In this context, heterogeneous cell–cell contact mimicking Notch‐inducing 3D hydrogels are expected to emerge as a valuable tool for the study of various tissue regeneration processes under highly reproducible conditions. In the future, it will become possible to design and fabricate hydrogels incorporating peptide ligands, signaling ligands, or other junctional adhesion molecules to create novel materials specifically aimed at generating the desired signaling for the ultimate application in regenerative medicine strategies.

It is important to acknowledge that, while the present study provided valuable insights into the potential of JAG1‐incorporated fibrin hydrogels in dental pulp regeneration, the obtained findings were primarily based on in vitro experiments and a subcutaneous implantation mouse model. Therefore, these findings have to be validated through additional in vivo studies using a relevant pre‐clinical large animal model of dental pulp injury and regeneration. Further, the process of fabricating these Notch‐signaling hydrogels and scaling up the hydrogel production has to be improved to facilitate the translation of these findings to clinical practice.

## 4 Conclusion

The Notch signaling hydrogel fabricated in the present study facilitated the differentiation of exogenous cells and promoted the paracrine functions of these cells, thereby enhancing tissue revascularization and angiogenesis. This feature enabled the creation of a microenvironment conducive to the regeneration of the pulp‐dentin complex. The prepared scaffold offers the advantages of a straightforward and controllable preparation process, which facilitates the regulation of Notch signaling intensity and achieving engineered cell seeding density. The Notch signaling hydrogel may, therefore, be considered as a safe, effective, and easily manipulable material for pulp tissue regeneration, with significant potential for clinical application.

## 5 Experimental Section

### Synthesis of JAG1 Immobilized Fibrin Gel

JAG1 immobilization within the fibrin gel was achieved as described in previous studies with certain modifications.^[^ ^36^, ^37^ ^]^ Briefly, sulfosuccinimidyl 4‐(N‐maleimidomethyl)cyclohexane‐1‐carboxylate (Sulfo‐SMCC, ThermoFisher, USA) was added to the fibrinogen stock solution, stirred for 10 min and then incubated at room temperature for 1 h. Subsequently, the reaction mixture was dialyzed in distilled water for 24 h in 3.5 kDa MW cutoff dialysis tube (Sigma) to obtain maleimide‐functionalized fibrinogen. Meanwhile, the Cys‐protein G (Beyotime) with a free cysteine at the N‐terminus was employed to capture Fc‐JAG1. 1 mg mL^−1^ Protein G was incubated with different concentrations of JAG1 at room temperature. The obtained solution was centrifuged at 1500 × g in a 30 kDa ultrafiltration tube (Millipore, USA) followed by washing with 3 times of PBS to remove the unbound protein G and concentrate cys‐protein G‐JAG1. The obtained cys‐protein G‐JAG1 was then added to the maleimide‐functionalized fibrinogen solution, mixed with the fibrin polymerization solution, and incubated at 37 °C for 1 h to prepare immobilized JAG1‐FIB (IJAG1‐FIB). Hydrogels that directly encapsulated soluble JAG1 (SJAG1‐FIB) or immobilized IgG Fc fragment (IIgG‐FIB) were used as control groups.

### Live/Dead Cell Viability Assay

JAG1 at various concentrations (0.25, 0.5, 1, 2, 3, 4 µg mL^−1^) was immobilized within FIB encapsulated with DPSCs at a concentration of 6 × 10^6^ mL^−1^. Cell viability was assessed using a live/dead cell staining kit (Invitrogen) according to the manufacturer's instructions. Briefly, the cells were incubated in 4 µM calcein AM at 37 °C for 30 min. After washing three times with PBS, the cells were stained with 4 µM propidium iodide (PI) at room temperature for 5 min. The live and dead cells were then imaged in the green channel (FITC/488 nm) and red channel (Texas Red/570 nm) as Z stacks under a confocal microscope (Olympus FV1200, Olympus, Japan). The percentage of viable cells was analyzed using Image J software.

### In Vitro Angiogenic Activities

DPSCs and HUVECs were mixed in equal proportions at a final concentration of 6 × 10^6^ cells mL^−1^ when encapsulated in gels with or without JAG1. The samples were cultured for 7 days in the absence of proangiogenic growth factors. Cell morphology, including angiogenic sprouting and tube formation, was characterized by immunostaining for CD31 (1:200, ab24590, Abcam) and visualized using two‐photon laser‐scanning microscopy (TPLSM, FluoView FV1000 MPE microscope, Olympus, Japan). Each sample was imaged at random positions using a z‐stack imaging volume of 500 µm × 500 µm × 80 µm. Four parameters, namely, the cell surface area, tubular volume, tubular length, and branch points, were quantified using Imaris software (Bitplane, UK), served as indicators of the capillary‐like structures.

### In Vitro Osteogenic Differentiation

The in vitro osteogenesis of the DPSCs and HUVECs co‐encapsulated in the gels was induced using the osteogenic medium (OM), which consisted of basal medium supplemented with 100 nM dexamethasone, 50 µg mL^−1^ ascorbic acid, and 5 mM sodium β‐glycerophosphate (all from Sigma), with or without JAG1. On Day 3 and Day 21 of the culture, the samples were fixed with 4% polyoxymethylene for 30 min and washed three times with PBS. Alkaline phosphatase (ALP) activity was assessed using the ALP staining assay. The samples were stained with a solution containing 0.25% naphthol AS‐BI phosphate and 0.75% Fast Blue BB (Sigma). The calcium deposits in the gels were analyzed through Alizarin Red S (ARS) staining (Sigma). The gels were stained with a 2% ARS solution at room temperature for 5 min. Images of the stained samples were acquired using a digital camera (Canon, Japan).

### RNA Isolation and RNA Sequencing Data Analysis

Total RNA was extracted using the TRIzol Reagent (Invitrogen) following the manufacturer's protocol. The genomic DNA was removed by DNase I (TaKara, Japan). RNA quality was determined by Agilent 2100 Bioanalyzer (Agilent Technologies, USA) and quantified using the ND‐2000 (NanoDrop Technologies, USA). Only the high‐quality samples with OD260/280≥1.8 and RNA integrity number > 8.0 were used in subsequent analysis. The mRNA and RNA sequencing processes were conducted to investigate the global expression profile of the fibrin gels, JAG1 immobilized gels, and soluble JAG1 encapsulated gels (n = 3), and 150–base pair paired‐end reads were generated. The adaptors and low‐quality reads from raw reads of each sample were trimmed to obtain clean reads. Then clean reads were separately aligned to the human reference genome GRCh38.p13 using the HISAT2 software. The mapped reads of each sample were assembled by StringTie in a reference‐based approach. The differential expression analysis was conducted using the DESeq2, genes with log2 fold change > 1.5 and P‐adjust ≤ 0.05 were considered differentially expressed. In addition, functional‐enrichment analysis including GO terms (Gene Ontology, http://www.geneontology.org) and KEGG pathways (Kyoto Encyclopedia of Genes and Genomes, http://www.genome.jp/kegg/) were carried out by Goatools (https://github.com/tanghaibao/Goatools) and KOBAS (http://kobas.cbi.pku.edu.cn/home.do).

### Short‐Term Subcutaneous Transplantation

All animal experiments described in this study were conducted in accordance with protocols approved by the Ethics Committee of West China Hospital of Stomatology, Sichuan University (ID: WCHSIRB‐D‐2022‐194). To assess the angiogenic differentiation capabilities, the DPSCs and human umbilical vein endothelial cells (HUVECs) co‐encapsulated gel samples including FIB, IIgG‐FIB, IJAG1‐FIB, and SJAG1‐FIB, were transplanted into the dorsum of the 6‐week‐old Balb/c nude mice obtained from Chengdu Dossy Experimental Animal Co. Ltd. All animal experiments conducted in this study were approved by the Institutional Animal Care and Use Committee of Sichuan University, and compliance with the ethical guidelines was ensured. The mice were housed in specific pathogen‐free facilities under standardized conditions. After 5 days of transplantation, the gels were harvested, fixed with 4% paraformaldehyde, and embedded in paraffin for use in the subsequent experiments.

### Long‐Term Semi‐In Situ Transplantation in Nude Mice

The odontogenic potential of the gels was evaluated using a semi‐in situ approach, in which the hydrogels combined with the treated dentin matrix (TDM) were transplanted into nude mice. TDM was utilized to create an inductive odontogenic environment for cell implants in vivo. The TDM used in the present study was obtained from swine mandible incisor teeth and prepared into several 5 mm‐length scaffolds. To demineralize the TDM scaffolds, they were subjected to gradually decreased concentrations of EDTA solution (from 17 to 5%) for 10 min, followed by three washes with deionized water for 5 min each. The prepared TDM was stored in PBS supplemented with penicillin (50 U ml^−1^) and streptomycin (50 mg ml^−1^) at 4 °C before use. The hydrogels were then seeded into the TDM canals, with one end of each canal sealed with iRoot BP Plus (Innovative BioCeramix Inc., Canada). Subsequently, the samples were transplanted into the left and right dorsum of nude mice for a duration of 6 weeks. Hematoxylin and eosin (H&E) and immunofluorescence staining were used to evaluate odontogenesis.

### H&E and Masson Staining

The harvested samples were fixed in 4% paraformaldehyde and then subjected to a 3‐month decalcification process using a 10% EDTA solution at pH 7.0. After decalcification, the samples were embedded in paraffin and sectioned into 5 µm thickness using a microtome (Leica, Germany). These tissue sections were deparaffinized in xylene and then rehydrated through a graded series of ethanol solutions. Subsequently, the sections were stained with H&E or Masson's trichrome (Beyotime) according to the manufacturer's instructions.

### Immunohistochemical Analysis and the Quantification of Vascular Density

Tissue sections were prepared for immunohistochemical staining following the same protocol as H&E staining. The primary antibody used was anti‐CD31 (1:100, sc‐376764, Santa Cruz). The secondary antibody used was anti‐mouse IgG‐Horseradish Peroxidase (HRP). The HPR‐conjugated secondary antibodies were detected using a 3,3′‐diaminobenzidine (DAB) kit (Beyotime). The subsequent steps were performed according to the manufacturer's instructions, and the immune reactions were visualized using a light microscope (Olympus). Vascular maturity was assessed by counting the CD31‐positive vessels with a diameter greater than 3 µm. The numbers of mature blood vessels with positive staining were normalized to the tissue area (mm^2^) by counting these vessels in 5 microscopic fields per sample.

### Immunofluorescence for Tissue Sections

Tissue sections were dewaxed, washed, and blocked before being incubated overnight at 4 °C with the following primary antibodies: anti‐DSPP (1:100, sc‐73632, Santa cruz), anti‐DMP1 (1:100, sc‐6551, Santa cruz), anti‐Collagen I (1:200, ab270993, Abcam), anti‐CD31 (1:200, ab24590, Abcam), anti‐VEGF (1:200, ab46154, Abcam), anti‐αSMA (1:200, ab5694, Abcam), anti‐NF200 (1:100, ab82259, Abcam), anti‐S100 beta (1:100, ab75478, Abcam). For detection, Alexa 555 goat anti‐rabbit (1:400, A21428, Invitrogen) and Alexa 488 goat anti‐mouse (1:400, A11001, Invitrogen) secondary antibodies were used. The sections were counterstained with DAPI and visualized using a confocal laser scanning microscope (Olympus FV1200, Olympus).

### Statistical Analysis

The data were presented as means ± standard deviations (SDs) from a minimum of three independent experiments. Statistical analysis was assessed by unpaired two‐tailed Student's t‐test to compare the two groups. Statistical significance was analyzed using SPSS 20.0 software (SPSS, USA). A value of P < 0.05 was considered statistically significant. *: P < 0.05, **: P < 0.01, ***: P < 0.001.

## Conflict of Interest

The authors declare no conflict of interest.

## Author Contributions

S.Z. performed conceptualization, methodology, investigation, formal analysis, wrote –original draft. M.Y. performed conceptualization, methodology, project administration, resources. M.L. performed investigation, methodology, formal analysis, wrote –review & edited the original draft. L.X. performed supervision, project administration. M.H. performed methodology, supervision, wrote –review & edited the original draft. F.H. performed methodology, funding acquisition, wrote –review & edited the original draft. W.T. performed supervision, funding acquisition, wrote –review & edited the original draft.

## Supporting information

Supporting Information

## Data Availability

The data that support the findings of this study are available from the corresponding author upon reasonable request.
