# Supplementary material for: Notch Signaling Hydrogels Enable Rapid Vascularization and Promote Dental Pulp Tissue Regeneration
Source: Adv Sci (Weinh). 2024 Jul 16;11(35):2310285. doi: 10.1002/advs.202310285 (PMC11425206; doi:10.1002/advs.202310285)
Supplement: Supplementary file 1 — Supporting Information [file ADVS-11-2310285-s001.pdf]

Supporting Information

Notch-inducing hydrogels enable rapid vascularization and promote dental pulp tissue regeneration

Siyuan Zhang, Mei Yu, Maojiao Li, Min He, Li Xie, Fangjun Huo *, Weidong Tian *

The Supplementary Materials Includes:

Supplementary Materials and Methods

S1. Cells culture

S2. Effects of IJAG1-FIB on monolayered cells

  S2.1. Cell proliferation assay

  S2.2. Cellular immunofluorescence

S3. Hydrogel fabrication and characterization

S3.1. Synthesis of fibrin gel

S3.2. Characterization of JAG1 immobilized hydrogel

S4. Enzyme-linked immunosorbent assay

S5. Reverse transcription-quantitative polymerase chain reaction (RT-qPCR)

S6. Western blot analysis

Supplementary Results

Supplementary Figures

Figure S1. Characterization of DPSCs.

Figure S2. Immunostaining assay to characterize HUVECs.

Figure S3. NOTCH1 and NOTCH3 expression in HUVECs and DPSCs with or without JAG1 treatment.

Figure S4. The biological safty inspection and evluation of IJAG1-FIB.

Figure S5. GSEA molecular feature of IJAG1-FIB.

Supplementary Tables

Table S1. Oligonucleotide primer sequences

Supplementary Materials and methods

S1. Cells culture

All cell isolation protocols were approved by the Ethics Committee of West China Hospital of Stomatology, Sichuan University (WCHSIRB-D-2021-015). Informed consent was obtained from all donors. DPSCs were isolated from the third molars of healthy individuals who underwent orthodontic or prophylactic therapies. Pulp tissues were separated from the teeth and immersed in a digestive solution containing 3 mg/mL collagenase I and 4 mg/mL dispase II for 1h at 37 °C. The isolated cells were then cultured in Alpha Modified Eagle's medium (α-MEM, Hyclone, USA) supplemented with 10% fetal bovine serum (FBS, Gibco, USA) and 1% penicillin/streptomycin solution. Their phenotypes were confirmed using flow cytometry, detecting surface markers including CD29, CD73, CD90, CD105, CD14, CD19, CD34, and CD45 (all from BD Biosciences, USA). HUVECs were isolated from human umbilical cord veins. The cord veins were rinsed with phosphate-buffered saline (PBS) and incubated with 1 mg/mL collagenase II for 30 min at 37 °C. The isolated cells were cultured in a complete endothelial cell medium (ECM, ScienCell, USA). Their phenotypes were confirmed by immunostaining for CD31, vWF, and αSMA. All cells were maintained at 37 °C in a humidified atmosphere with 5% CO_2, and the culture medium was changed every other day. For all experiments, cells between passages 2 and 6 were used.

S2. Effects of IJAG1-FIB on monolayered cells

S2.1. Cell proliferation assay

The cell viability of DPSCs or HUVECs cultured on IJAG1-FIB was assessed using a Cell Counting Kit-8 (CCK-8, Beyotime, China). DPSCs or HUVECs were seeded at a density of 500 cells/well in 96-well plates precoated with hydrogels and maintained in a growth medium. On days 1, 3, 5, and 7, the cells in each well were incubated with 10 μl of WST-8 solution for 2 h at 37 °C. The absorbance was then measured at 450 nm using a microplate reader (BioTek, USA).

S2.2. Cellular immunofluorescence

DPSCs or HUVECs were seeded on coverslips precoated with hydrogels in 6-well plates. After two days of culture, the cells were washed three times with PBS and fixed with 4% paraformaldehyde at room temperature for 30 min. Fixed cells were then

permeabilized with 0.5% Triton X-100 for 10 min (permeabilization was not used for cell surface antigens) and subsequently blocked in 10% normal goat serum for 1 hour at room temperature. The cells were incubated with primary antibodies, including rabbit anti-vWF (1:100, ab7356, Millipore), rabbit anti-VEGFA (1:100, ab46154, Abcam), and mouse anti-αSMA (1:100, ab5694, Abcam) antibodies, followed by washing and incubating with secondary anti-rabbit conjugated Alexa Fluor 488 (1:400, A11008, Invitrogen) and anti-mouse conjugated Alexa Fluor 555 (1:400, A21422, Invitrogen) antibodies. Cells were lastly counterstained with DAPI and visualized using a fluorescence microscope (Zeiss, Germany).

S3. Hydrogel fabrication and characterization

S3.1. Synthesis of fibrin gel

Human fibrinogen (Sigma) was dissolved in 0.9% NaCl and incubated at 37 °C for 60 min. The concentration of fibrinogen after sterile filtration was determined by measuring absorbance at 280 nm using a spectrophotometer (BioTek). Fibrin gels (FIB) were polymerized by adding thrombin solution (from bovine plasma, Sigma) and allowed to gel for 30 min at 37 °C. The final fibrin hydrogel contained 5 mg/mL fibrinogen, 2 U/mL thrombin, 20 mM calcium chloride ($CaCl_2$, Sigma). For cell encapsulation, the prepared cell suspensions, thrombin, and $CaCl_2$ were thoroughly mixed with fibrinogen stock solution.

S3.2. Characterization of JAG1 immobilized hydrogel

The mechanical property of the synthesized hydrogels was determined using a stress-controlled rheometer (ThermoFisher, USA). Cell-free samples of FIB, IJAG1-FIB, and SJAG1-FIB were subjected to rheological characterization by measuring their elastic modulus, which serves as an indicator of gel stiffness. The rheological measurements were performed at 37 °C using a parallel plate (20 mm) setup. The contribution of the storage modules (G'), representing solid-like behavior, and the loss modulus (G''), representing liquid-like behavior, were recorded. The rheometer was operated in a frequency sweep mode, with frequencies ranging from 0.1 to 10 Hz, under a constant 1% shear strain.

The biodegradation and swelling behavior of cell-free hydrogels were evaluated over 4 weeks under sterile conditions. The *in vitro* degradation rate was assessed by

immersing pre-weighed hydrogels (W0) in PBS containing 2.4 nM plasmin (Sigma), according to a previous study [1]. The hydrogels were incubated at 37 °C for various time points. On each measurement day, the samples were lyophilized and re-weighed to obtain their respective dry weights (Wt). The pH value of the surrounding PBS was also measured. The percentage degradation was calculated using the following equation:

Percentage degradation = [(W0-Wt)/W0]×100

Hydrogel swelling analysis was conducted at a neutral pH and 37 °C. The gel samples were prepared on day 0 and weighed (W0). They were then submerged in PBS and incubated. On each measurement day, the hydrogels were weighed (Wt′) after removing excess PBS. The swelling ratio was calculated using the following equation:

Swelling ratio = Wt′/W0

The microstructure of the synthesized hydrogels was analyzed using scanning electron microscopy (SEM). FIB or IJAG1-FIB samples were fixed in 3% glutaraldehyde, washed with PBS, and then dehydrated in a series of solutions with increasing proportions of acetone. Subsequently, the gel samples were dried using critical-point drying with CO_2 and sputter-coated with a 20 nm layer of gold-palladium. Images of the samples were acquired using an ESEM XL 30 FEG microscope (FEI/Philips, USA).

The immobilization of JAG1 was examined using a specific antibody. FIB, IIgG-FIB, IJAG1-FIB, and SJAG1-FIB samples were prepared and kept in PBS for three days. The gel samples were then incubated with a primary rabbit anti-Jagged1 antibody (1:100, ab7771, Abcam), followed by washing and incubating with a secondary anti-rabbit antibody conjugated to Alexa Fluor 488 (1:400, Invitrogen). The gels were visualized using a confocal microscope (Olympus FV1200, Olympus) to observe the fluorescence signals indicating the presence of JAG1.

S4. Enzyme-linked immunosorbent assay

Secretion levels of VEGFA, PDGF-BB, and ANG1 proteins in gel supernatants were quantified using enzyme-linked immunosorbent assay (ELISA) kits (Elabscience, USA) according to the manufacturer's instructions. Briefly, standards and gel supernatants were added to antibody-precoated ELISA plate wells and incubated for 90 min at 37 ℃. Subsequently, the plates were incubated with biotinylated detection

solution, HRP conjugates, and substrate Reagent in sequential steps. The absorbance of each well was measured at 450 nm using a microplate reader (BioTek). Cumulative amounts of VEGFA, PDGF-BB, and ANG1 secretion were calculated based on a standard curve (n = 3).

S5. Reverse transcription-quantitative polymerase chain reaction (RT-qPCR)

Total RNA was isolated from cells or gel samples by TRIzol Reagent (Invitrogen) following the manufacturer's instructions (n = 5). The expression levels of various genes were determined by quantitative polymerase chain reaction (qPCR). Notch signaling-related genes, including JAG1, Dll4, Notch1, Notch2, Notch3, Hes1, and Hey1, were evaluated. Angiogenesis-related genes, including CD31, CD34, VE-cadherin, and αSMA, were analyzed. Osteogenesis and odontogenesis-related genes, such as ALP, Runx2, OPN, COL1A1, and DMP1, were also examined. RNA was reverse transcribed into complementary DNA (cDNA) using the HiScript III 1st Strand cDNA Synthesis Kit (Vazyme, China). Real-time qPCR was performed on a QuantStudio 6 Flex System (Applied Biosystems, USA) using SYBR Green PCR mix (ThermoFisher). The expression of each gene was normalized to GAPDH and analyzed using the relative quantification $2^{-\triangle\triangle}$ Ct method. Primer sequences can be found in Table S1 in the Supplementary Materials.

S6. Western blot analysis

Gel samples, collected either in vitro or in vivo, were lysed in RIPA buffer supplemented with a protease inhibitor cocktail (Millipore). The protein concentrations were determined using a BCA Protein Assay (Thermo Fisher Scientific). Protein extracts were subjected to SDS-polyacrylamide gel electrophoresis and transferred onto polyvinylidene fluoride (PVDF) membranes. The membranes were blocked in 5% skim milk and incubated overnight at 4 ℃ with primary antibodies, including CD31 (1:1000, sc-376764, Santa Cruz), CD34 (1:1000, sc-7324, Santa Cruz), αSMA (1:1000, ab5694, Abcam), PDGFRβ (1:1000, 383331, Zen Bioscience), VEGFR2 (1:1000, 222820, Zen Bioscience), and Actin (1:5000, 200068-8F10, Zen Bioscience). After thorough washing, the membranes were incubated with HRP-conjugated secondary antibodies. Protein bands were visualized using an electrochemiluminescence (ECL) Western Blotting Substrate reagent (ThermoFisher), and images were captured using a

chemiluminescence detector (ImageQuant LAS 4000 Mini, GE Healthcare, USA). The intensity of bands was quantified using ImageJ software and normalized to GAPDH.

Supplementary Results

S1. Isolated cell characterization

DPSCs were characterized by a fibroblast-like morphology (Figure S1A). The results of multi-differentiation induction assays indicated that DPSCs were capable of osteogenic (Figure S1B), adipogenic (Figure S1C), and chondrogenic differentiation (Figure S1D). In addition, DPSCs were characterized based on surface marker expression following the minimal criteria by the International Society of Cellular Therapy to define MSCs [2]. DPSCs demonstrated robust expression (>99%) of the mesenchymal markers CD29, CD73, CD90, and CD105, while showing minimal expression (<1%) of the hematopoietic lineage markers CD14, CD19, CD34, and CD45 (Figure S1E). These findings confirmed that the isolated cells were dental pulp mesenchymal stromal cells.

HUVECs were characterized using immunostaining techniques. The HUVECs exhibited a characteristic "cobblestone" morphology and displayed positive staining for CD31, vWF, which are specific markers for endothelial cells (Figure S2A). Moreover, the cells demonstrated negative expression for αSMA, a marker associated with pericytes (Figure S2B). These results indicate the successful isolation of endothelial cells from human umbilical cords, suggesting the purity and identity of the HUVECs.

Supplementary Figures

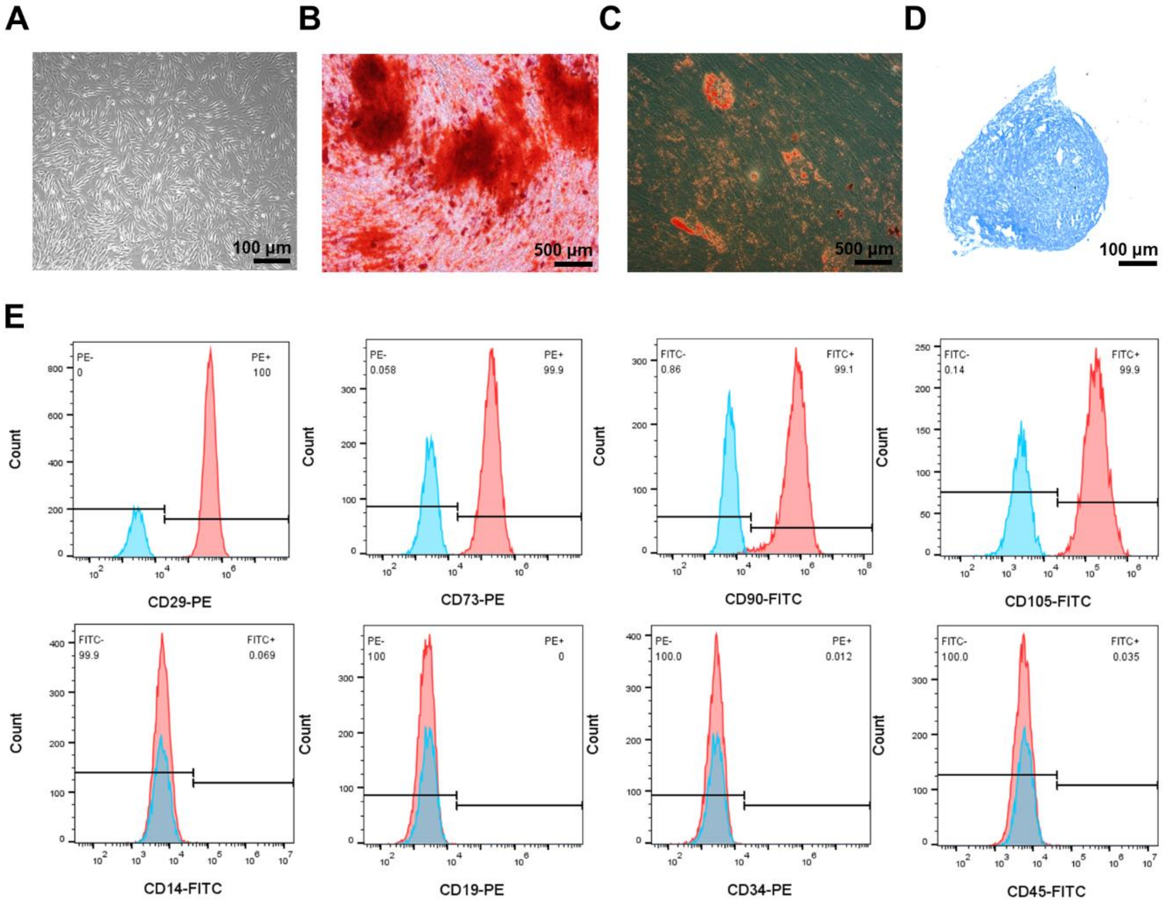

Figure S1. Characterization of DPSCs. A) The typical morphology of mesenchymal stem cells with spindle shapes was observed in DPSCs. B-D) Detection of multi-differentiation of DPSCs. B) Osteogenic differentiation. Representative images of alizarin red s staining of DPSCs with mineral nodules after osteogenic culturing for 14 days. C) Adipogenic differentiation. Representative images of oil red o staining of DPSCs with lipid clusters after adipogenic induction for 21 days. D) Cartilage differentiation. Representative images of toluidine blue staining of DPSCs with round-shaped cell clusters after chondrogenic differentiation for 21 days. E) Immunophenotype analysis of DPSCs by flow cytometry. DPSCs showed positive expression for mesenchymal markers (CD29, CD73, CD90, and CD105) and negative expression for hematopoietic lineage markers (CD14, CD19, CD34, and CD45).

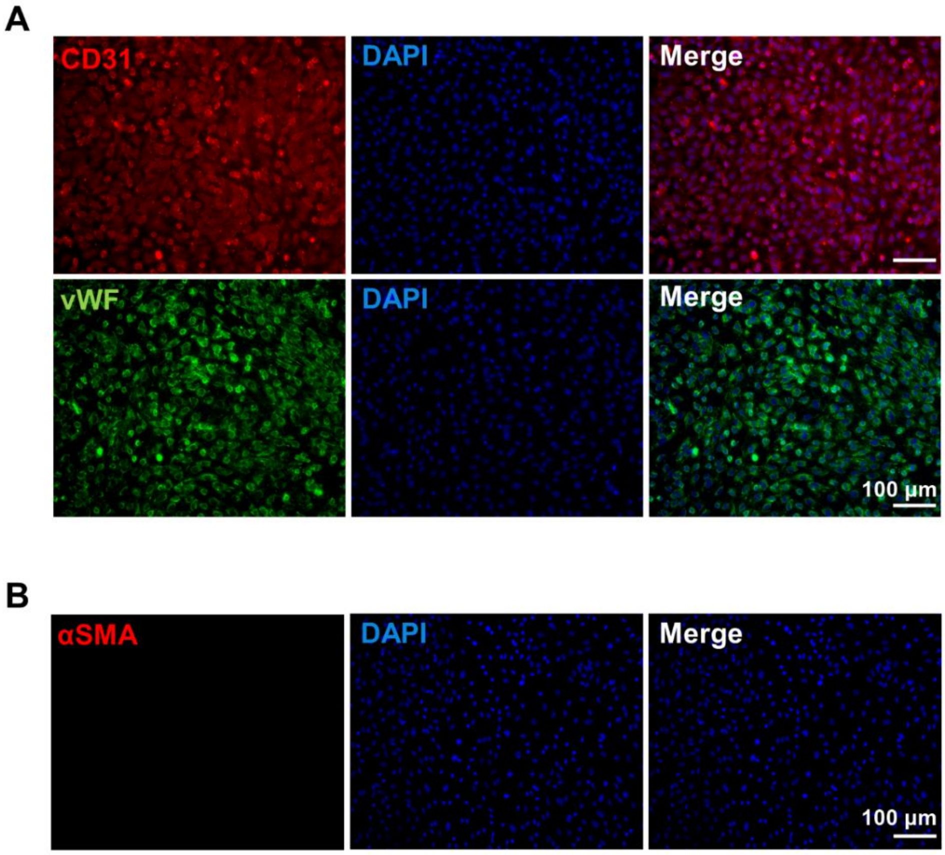

Figure S2. Immunostaining assay to characterize HUVECs. A) HUVECs exhibited positive staining for endothelial markers (CD31 and vWF). B) HUVECs showed negative staining for the pericyte marker (αSMA).

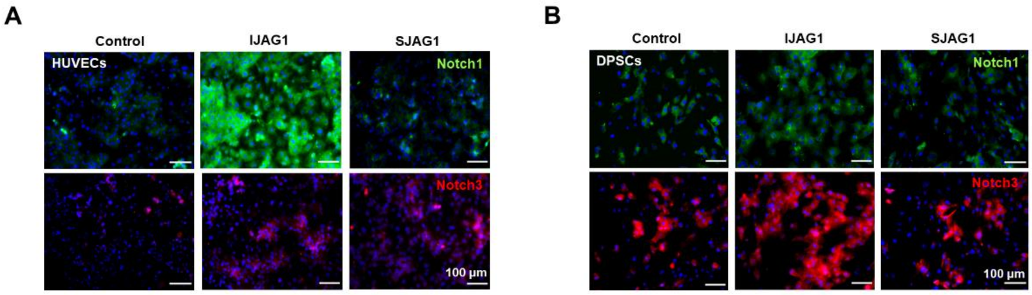

Figure S3. NOTCH1 and NOTCH3 expression in A) HUVECs and B) DPSCs with or without JAG1 treatment.

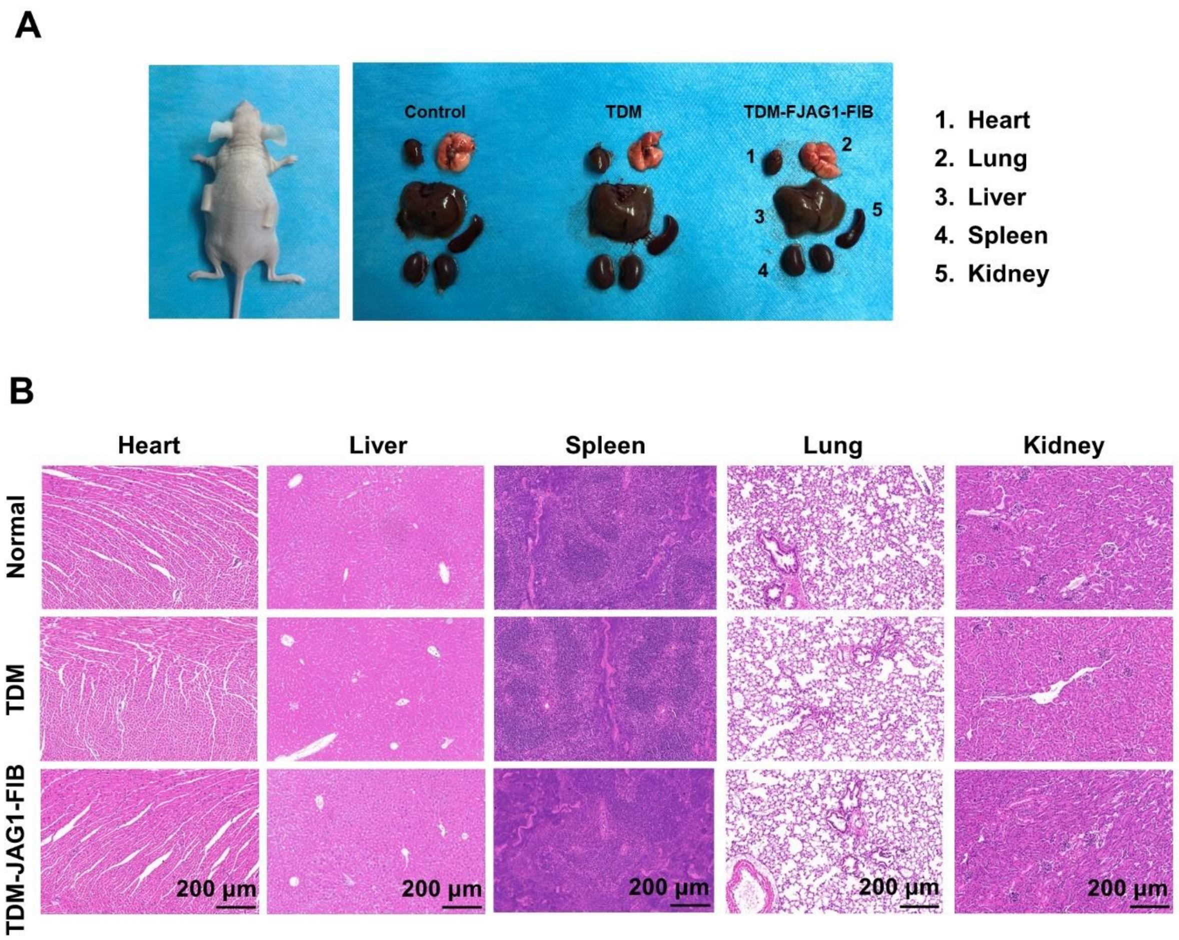

Figure S4. The biological safety inspection and evaluation of IJAG1-FIB. A) The major organs of the nude mice were dissected and evaluated. B) HE-stained sections of the heart, liver, spleen, lung, and kidney of the nude mice in the experimental groups showed no noticeable differences, verifying the biocompatibility of the IJAG1-FIB in vivo.

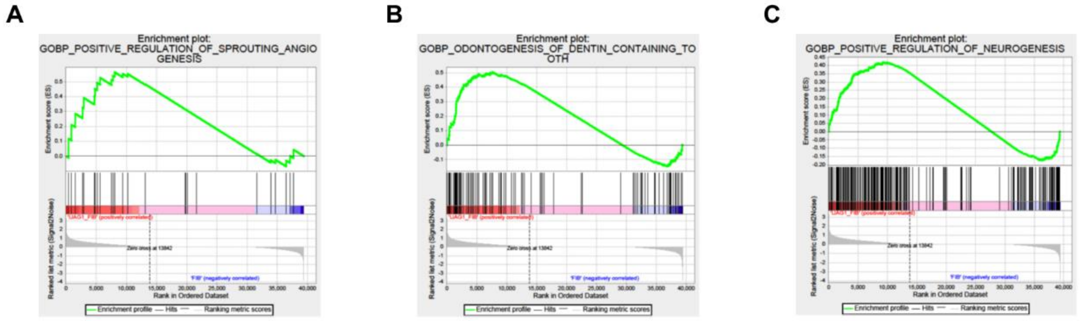

Figure S5. GSEA molecular feature of IJAG1-FIB. The positive regulation of gene sets of A) POSITIVE_REGULATION_OF_SPROUTING_ANGIOGENESIS, B) ODONTOGENESIS_OF_DENTIN_CONTAINING_TOOTH, and C) POSITIVE_REGULATION_OF_NEUROGENESIS are significantly enriched in IJAG1-FIB.

Supplementary Tables

Table S1. Oligonucleotide primer sequences

Gene name	Forward (5’-3’)	Reverse (5’-3’)
GAPDH	GGAAGCTTGTCATCAATGGAAATC	TGATGACCCTTTTGGCTCCC
DLL4	GGGTCAGAACTGGTTATTGGATG	TGACAGCCCGAAAGACAGATAG
JAG1	GCCAGGAAGTTTCAGGGAGA	GCTGGAGACTGGAAGACCGA
NOTCH1	ACTGCTCCCTCAACTTCAATGAC	ACTGGTCGTACAGGGGGTTG
NOTCH2	CTGAACGATGGTCGCTGCA	TGCCCTGTGAATCCTGGTGA
NOTCH3	GCTTGGGAAATCAGCCTTACACT	CTGTCCAGGTGGTCGGTGAT
HES1	ATTCTGGAAATGACAGTGAAGCAC	CACCTCGGTATTAACGCCCTC
HEY1	GGCAGGAGGGAAAGGTTACTT	GAAGCGTAGTTGTTGAGATGCG
BSP	GAACCACTTCCCCACCTTTT	TCTGACCATCATAGCCATCG
RUNX2	CTTTACTTACACCCCGCCAGTC	AGAGATATGGAGTGCTGCTGGTC
OPN	CAGTTGTCCCCACAGTAGACAC	GTGATGTCCTCGTCTGTAGCATC
COL1A1	AACATGGAGACTGGTGAGACCT	CGCCATACTCGAACTGGAATC

DSPP	CGACATAGGTCACAATGAGGATGTCG	TTGCTTCCAGCTACTTGAGGTC
DMP1	CTCGCACACACTCTCCCACTCAAA	TGGCTTTCCTCGCTCTGACTCTCT
WNT3	TGACTCGCATCATAAGGGGC	GTGGTCCAGGATAGTCGTGC
WNT5A	CTGCAGCCAACTGGCAGGACT	CGCGGCTGCCTATCTGCATCA
DKK1	CTCCGGTCATCAGACTGTGC	CCGGCAAGACAGACCTTCTC
LEF1	AAATAAAGTGCCCGTGGTGC	GAGCTGGAGGATGTCTGGAC
CCN4	GAAGCAGTCAGCCCTTATG	CTTGGGTGTAGTCCAGAAC

References

[1] K. M. Lorentz, S. Kontos, P. Frey, J. A. Hubbell, *Biomaterials* 2011, 32, 430.

[2] M. Dominici, K. Le Blanc, I. Mueller, I. Slaper-Cortenbach, F. Marini, D. Krause, R. Deans, A. Keating, D. Prockop, E. Horwitz, *Cytotherapy* 2006, 8, 315.
